# Multi-strain probiotics attenuate carbohydrate-lipid metabolic dysregulation in type 2 diabetic rats via gut-liver axis modulation

**DOI:** 10.1128/msystems.00369-25

**Published:** 2025-06-10

**Authors:** Yecheng Gao, Yanfang Liu, Zelong Li, Liyi Lu, Yajuan Guo, Dun Su, Heping Zhang

**Affiliations:** 1 Inner Mongolia Key Laboratory of Dairy Biotechnology and Engineering, Inner Mongolia Agricultural University 117454 , Hohhot, Inner Mongolia, China; 2 Key Laboratory of Dairy Products Processing, Ministry of Agriculture and Rural Affairs, Inner Mongolia Agricultural University 117454 , Hohhot, Inner Mongolia, China; 3 Key Laboratory of Dairy Biotechnology and Engineering, Ministry of Education, Inner Mongolia Agricultural University 117454 , Hohhot, Inner Mongolia, China; 4 Perfect (Guangdong) Co., Ltd. 722412 , Zhongshan, Guangdong, China; APC Microbiome Ireland, Cork, Ireland

**Keywords:** type 2 diabetes, probiotics, gut microbiota, transcriptome, short-chain fatty acids, bile acids

## Abstract

Type 2 diabetes mellitus (T2DM) is a pervasive chronic metabolic disorder characterized by dysregulation of carbohydrate, protein, and lipid metabolism. The objective of this study was to elucidate the impact of multi-strain probiotic supplementation on the metabolism of carbohydrates and lipids and the interplay between the gut and liver in a T2DM rat model. A total of 32 rats were randomly assigned to four experimental groups: a control group, a T2DM model group, a low-dose probiotics group, and a high-dose probiotics group. The impact of probiotic intervention on glycemic and lipid profiles was evaluated, with a specific emphasis on the high-dose cohort. The treatment with multi-strain probiotics, consisting of three *Lacticaseibacillus* species and one *Bifidobacterium* species, resulted in a significant improvement in blood glucose and lipid profiles in T2DM rats, with the highest dosage demonstrating the most pronounced effects. Probiotic administration modulated gut microbiota composition and diversity, enriching potentially beneficial bacterial species and altering gut metabolic modules and carbohydrate-active enzyme profiles. Multi-omics analyses indicated that alterations in fecal short-chain fatty acids and serum bile acids may serve as pivotal mediators for crosstalk between hepatic and gut transcriptomic pathways. This study offers novel insights into the role of probiotics in managing T2DM via the gut-liver axis, emphasizing the potential of probiotic therapy in modulating key metabolic pathways.

**IMPORTANCE:**

Type 2 diabetes mellitus (T2DM) is a chronic metabolic disease characterized by hyperglycemia, caused by defects in insulin secretion, insulin action, or both. For individuals diagnosed with T2DM, managing diabetes-related complications is often the most challenging aspect. Exogenous probiotics have the potential to serve as a promising therapeutic strategy to improve diabetes-related symptoms. We conducted a 64-day animal experiment to investigate the effects of probiotics on T2DM-related metabolic disorders and dyslipidemia by feeding four mixed probiotics to T2DM rats. The results showed that probiotics exerted beneficial effects on glucose- and lipid-related homeostasis indices in diabetic rats to some extent and modulated the gut microbiota to manage T2DM via the gut-liver axis.

## INTRODUCTION

Type 2 diabetes mellitus (T2DM), a chronic metabolic disorder driven by insulin resistance and β-cell dysfunction, affects over 537 million adults globally, posing urgent challenges for sustainable management strategies (1, 2). While medications like metformin and glucagon-like peptide-1 agonists (e.g., liraglutide) effectively lower hyperglycemia, their widespread use is hampered by gastrointestinal adverse effects and nutrient deficiencies (3–6). These limitations underscore the need for adjunctive approaches targeting the multifactorial pathogenesis of T2DM, particularly its systemic complications linked to advanced glycation end-products and metabolic dysregulation (3).

Emerging evidence implicates gut microbiota dysbiosis as a central player in T2DM progression through disrupted host-microbe crosstalk (7, 8). The gut microbiome, often termed the “second genome” (9, 10), modulates critical metabolic pathways via short-chain fatty acid (SCFA) production, bile acid metabolism, and immune-inflammatory signaling (11, 12). Notably, diabetic dyslipidemia, marked by elevated triglycerides and atherogenic low-density lipoprotein (LDL) particles, exacerbates insulin resistance through impaired glucose uptake and chronic inflammation (13, 14). Mechanistically, microbiota-derived SCFAs and bile acids regulate glucose homeostasis by activating enteroendocrine receptors (e.g., farnesoid X receptor, G protein-coupled bile acid receptor) along the gut-liver axis, while dysbiosis disrupts this interplay, aggravating metabolic dysfunction (15–17). This bidirectional relationship positions gut microbiota modulation as a promising therapeutic frontier.

Probiotic interventions have demonstrated potential in restoring microbial balance and ameliorating diabetic phenotypes (18–21). Specific lactobacilli and bifidobacterial strains have been shown to improve insulin sensitivity by enhancing SCFA production, suppressing α-glucosidase activity, and reducing systemic inflammation (22–25). However, therapeutic efficacy is strain- and disease-dependent, with variable outcomes reported across clinical and preclinical studies (26, 27). For instance, while single-strain probiotics, such as different *L. rhamnosus* strains, have been shown to improve glucose homeostasis in various rodent models (28–30), multispecies formulations may offer synergistic benefits through complementary mechanisms (31, 32). This underscores the need for rationally designed probiotic combinations tailored to T2DM-specific metabolic perturbations.

Building on these insights, we hypothesized that a multispecies probiotic consortium targeting key gut-liver axis pathways could mitigate T2DM-associated dysglycemia and dyslipidemia. We evaluated a formulation comprising *Limosilactobacillus fermentum* LFPerfectus001, *Lacticaseibacillus rhamnosus* LRPerfectus158, *Lactiplantibacillus plantarum* LPPerfectus001, and *Bifidobacterium animalis* subsp. *lactis* BAPerfectus006, strains selected for their roles in SCFA production, bile acid metabolism, and anti-inflammatory activity (33–36). Using a diabetic rat model, we investigated the consortium’s effects on glycemic control and lipid profiles, its influence on gut microbiota structure and SCFA levels, and its modulation of bile acid content along the gut-liver axis. This work advances the translational potential of precision probiotics in T2DM management by elucidating strain-specific and synergistic mechanisms.

## RESULTS

### Probiotics relieve diabetes symptoms

**Fig 1:**
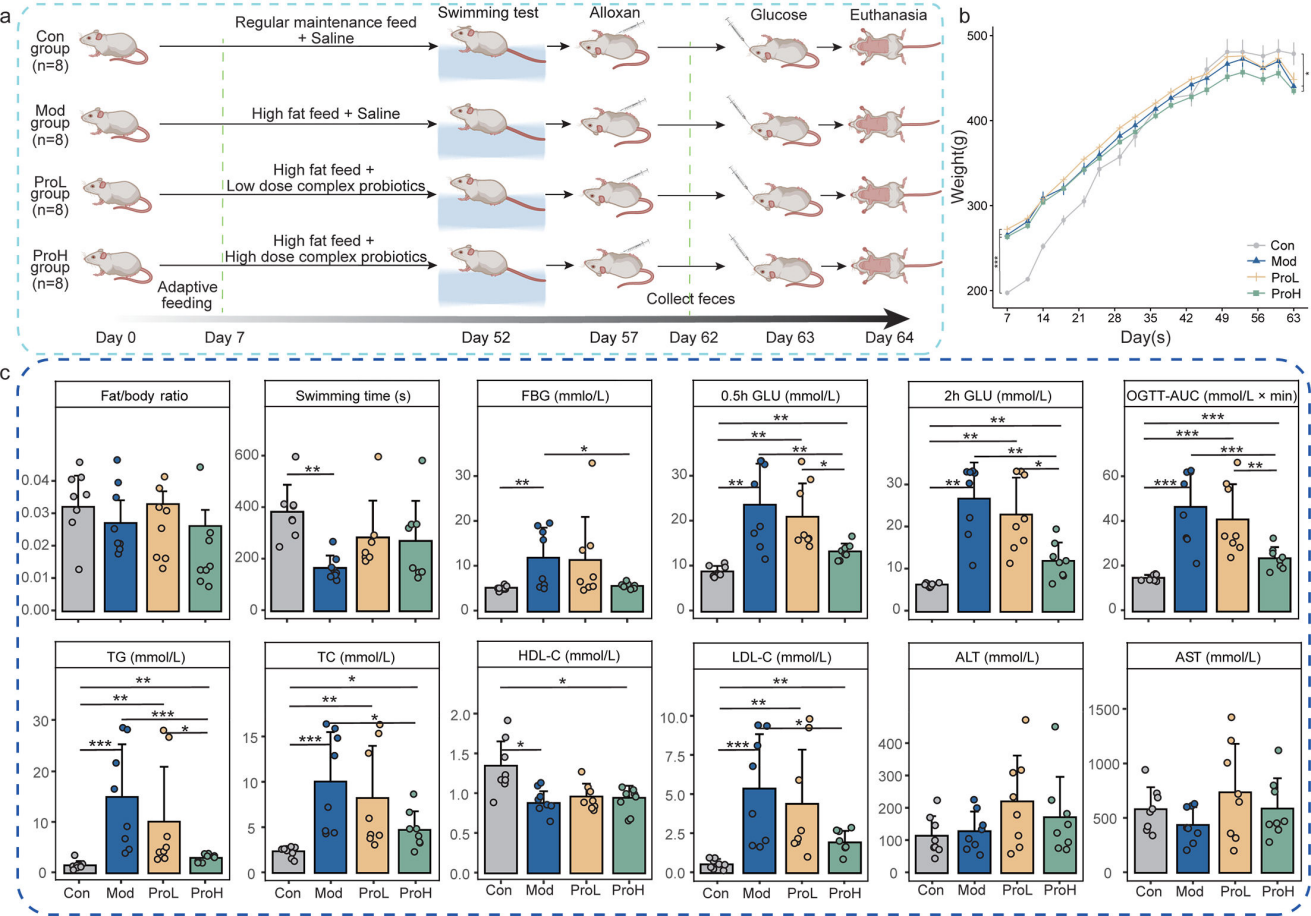
Experimental design and probiotic effects on metabolic and physiological parameters in T2DM rats. (**a**) Schematic timeline of probiotic interventions and sample collection. (**b**) Body weight changes across experimental groups from adaptation phase completion to intervention endpoint. (**c**) Post-intervention group differences in body composition, exercise capacity, glycemic, and lipid indices. Data are presented as means ± SEM (*n* = 8). Significance levels: *P < 0.05, **P < 0.01, ***P < 0.001.

Following a 7-day period of high-fat chow feeding and the exclusion of obesity-resistant rats (Fig. 1a), no significant difference in body weight was observed between the model and probiotic groups. However, both groups exhibited significantly higher body weights than the control group (Con) (*P* < 0.01; Fig. 1b). Notably, a decrease in body weight was evident after the probiotic intervention. Furthermore, the administration of probiotics did not significantly affect the lipid/body weight ratio of rats (Fig. 1c). To assess the effect of the diabetes model on the swimming endurance of rats, a weight-bearing swimming test was conducted on day 52. The probiotic intervention increased swimming time among the modeled rats; however, none of them exhibited a recovery to the level observed in the control group (Fig. 1c).

On day 63, after a 10 to 12 h fasting period, an oral glucose tolerance test (OGTT) was administered to evaluate glucose tolerance in the rats. As shown in Fig. 1c, compared to the model (Mod) group, low-dose probiotics (ProL) slightly reduced the fasting blood glucose (FBG) and the glucose levels at 0.5 and 2 h post-gavage. The area under the curve (AUC) of the oral glucose tolerance test showed similar results. Conversely, the high-dose probiotics (ProH) significantly reduced glucose levels at 0.5 and 2 h post-gavage, as well as the AUC of the oral glucose tolerance test (*P* < 0.01; Fig. 1c; Table S1). These findings suggest that probiotics are capable of improving glucose regulation in diabetic rats.

In addition, the levels of TG, TC, and LDL-C were significantly elevated in diabetic rats, but these levels showed a notable reduction following probiotic supplementation, particularly in the ProH group (*P* < 0.05; Fig. 1c), and approached those observed in the Con group. Meanwhile, HDL-C levels showed no significant difference between the Mod group and the two probiotic groups. No statistically significant differences were observed in serum alanine aminotransferase (ALT) and aspartate aminotransferase (AST) levels between the groups (*P* > 0.05; Fig. 1c). These results indicate that probiotics may exert a beneficial effect on blood glucose and lipid-related homeostasis indexes in diabetic rats to some extent.

### Probiotics regulate the composition of gut microbiota in diabetic rats

**Fig 2:**
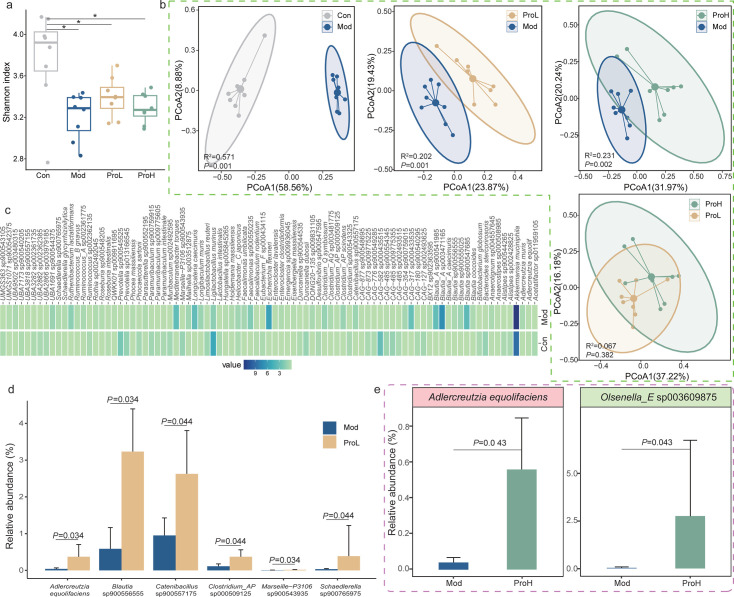
Gut microbiota diversity and species-level genome bin (SGB) features in T2DM rats. (**a**) Shannon diversity indices of gut microbiota in control (Con), model (Mod), low-dose probiotic (ProL), and high-dose probiotic (ProH) groups. (**b**) PCoA score plots of β-diversity, with Adonis test results shown in the lower left corner. (**c**) Heatmap of SGBs showing significant differential abundance between Mod and Con groups post-modeling (green: reduced, blue: enriched). (**d and e**) SGBs with significant differential abundance between the specified groups. Data are presented as means ± SEM (*n* = 8); *P < 0.05.

On day 62, shotgun metagenomic sequencing was employed to analyze the fecal microorganisms of rats. A significant reduction in alpha diversity (expressed as the Shannon diversity index) was observed in rats after modeling, compared to the Con group (Fig. 2a; *P <* 0.05; Wilcoxon test). Following intervention with probiotics, a recovery trend was observed; however, no statistically significant difference was noted when compared to the Mod group. Similarly, beta diversity (principal coordinate analysis [PCoA] and Adonis analysis) was observed between the Con group and the Mod group (Fig. 2b; *R*^2^ = 0.571, *P* = 0.001), the Mod group and the ProL group (Fig. 2b; *R*^2^ = 0.202, *P* = 0.001), and the Mod group and the ProH group (Fig. 2b; *R*^2^ = 0.231, *P* = 0.002). However, this was not observed between the ProL group and the ProH group (Fig. 2b; *R*^2^ = 0.067, *P* = 0.382).

Subsequently, the fecal microbiota of rats was analyzed at the species level, resulting in the identification of 174 species-level genome boxes (SGBs) across all samples. Of these, 109 were classified as major SGBs with a relative abundance of >0.1% (Table S2). To obtain a more detailed understanding of the specific changes in the gut microbiota following modeling and probiotic intervention, the non-parametric Wilcoxon rank sum test was applied to calculate the differential SGBs between groups. A total of 93 different SGBs were identified after modeling (Fig. 2c; Table S3). After probiotic intervention, a significant increase in the number of *Adlercreutzia equolifaciens*, *Blautia* sp900556555, *Clostridium*_AP sp000509125, *Catenibacillus* sp900557175, *Marseille*-P3106 sp900543935, and *Schaedlerella* sp900765975 was observed in the low-dose group (Fig. 2d, corrected *P* < 0.05; Wilcoxon test). Furthermore, the number of *Adlercreutzia equolifaciens* and *Olsenella*_E sp003609875 in the high-dose group increased significantly (Fig. 2e, corrected *P* < 0.05; Wilcoxon test). It is noteworthy that, regardless of probiotic dosage, *Adlercreutzia equolifaciens* increased significantly in comparison to the Mod group, thereby substantiating the assertion that probiotics possess the capacity to regulate the composition and structure of the intestinal microbiota in rats.

### Probiotics modulate the gut metabolic modules and carbohydrate-active enzymes in rats

**Fig 3:**
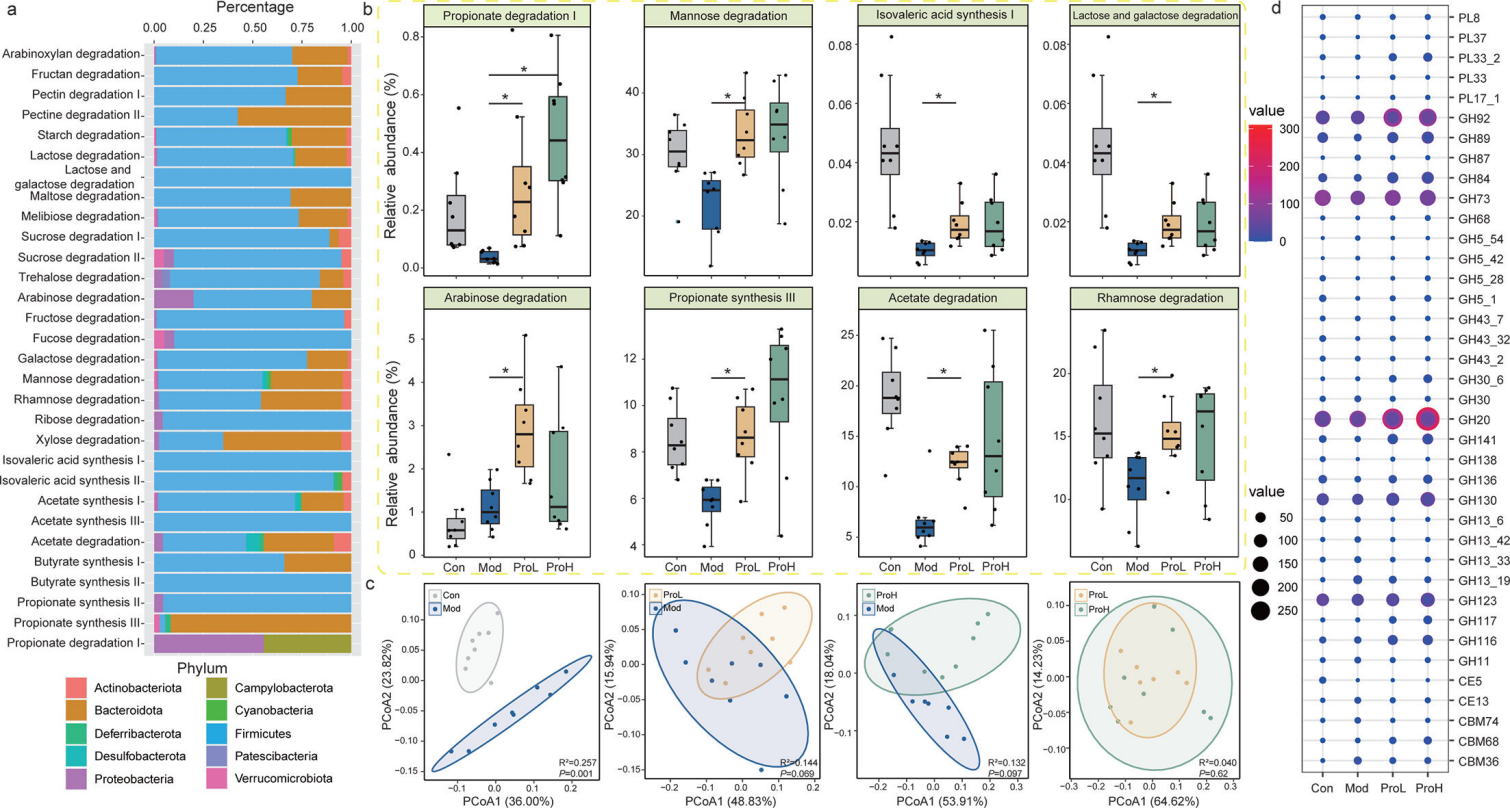
Changes in GMM and carbohydrate-active enzyme (CAZyme) profiles in the Con, Mod, ProL, and ProH groups during the trial. (**a**) Distribution of GMMs in T2DM rats. Different color blocks represent different phyla. (**b**) Responsive GMMs showing differences after probiotic intervention. (**c**) PCoA score plot showing changes in the predicted CAZyme profiles of the Con, Mod, ProL, and ProH groups. (**d**) Bubble plot showing the significant differences in CAZyme subfamilies with higher gene accumulation after probiotic intervention. PL, polysaccharide lyases; GH, glycoside hydrolases; CE, carbohydrate esterases; CBM, carbohydrate-binding modules. Data were presented as the means ± SEM (*n* = 8). *P < 0.05.

The functional differences among the different groups were revealed through an analysis of the gut metabolic modules (GMMs) encoded by the gut microbiota. A total of 30 GMMs were identified, belonging to 10 different phyla (Fig. 3a). The majority were classified as Firmicutes (67.4%), followed by Bacteroidota (20.5%) and Actinobacteriota (4.0%). Interestingly, after the intervention, a comparison of the cumulative abundance of GMMs encoded by SGBs between the groups revealed an increase in the propionate degradation I, mannose degradation, isovalerate synthesis I, lactose and galactose degradation, arabinose degradation, propionate synthesis III, acetate degradation, and rhamnose degradation pathways following probiotic intake (Fig. 3b). These findings indicate that these GMMs may be instrumental in the progression of T2DM.

To explore the enzyme repertoire utilized for complex polysaccharide metabolism, we undertook a comprehensive analysis of genes encoding carbohydrate-active enzymes (CAZymes) present in the rat fecal microbiome. A total of 15,358 CAZyme-encoding genes were identified in 189 SGBs (Table S4), with the majority of genes encoding glycoside hydrolase families (GHs; 8,498 genes), followed by glycosyltransferases (GTs; 4,007 genes) and carbohydrate esterases (CEs; 1,676 genes). The diversity of CAZymes among the groups was evaluated by comparing the cumulative abundance, which was calculated based on the distribution of CAZymes in SGBs. PCoA analysis of the CAZyme profile showed a comparable trend of change in the gut microbiota, with significant differences between the Con group and the Mod group (*R*^2^ = 0.257, *P* = 0.001; Fig. 3c). Apparent clustering patterns were also found between the Mod group and the ProL group (*R*^2^ = 0.144, *P* = 0.069; Fig. 3c), the Mod group and the ProH group (*R*^2^ = 0.132, *P* = 0.097; Fig. 3c), but not between the ProL group and the ProH group (*R*^2^ = 0.040, *P* = 0.62; Fig. 3c). Subsequently, the CAZymes that exhibited differential expression in response to probiotic intervention were identified, revealing 38 significantly altered CAZymes, predominantly from the polysaccharide lyase (PLs) and GHs families. The cumulative abundance of genes in 24 CAZyme families was significantly higher than that in the Mod group, while the remaining 14 displayed the opposite trend (Fig. 3d). These findings indicate that probiotics may play a role in the regulation of carbohydrates in the rat gut.

### Probiotics modulate key metabolites in diabetic rats

**Fig 4:**
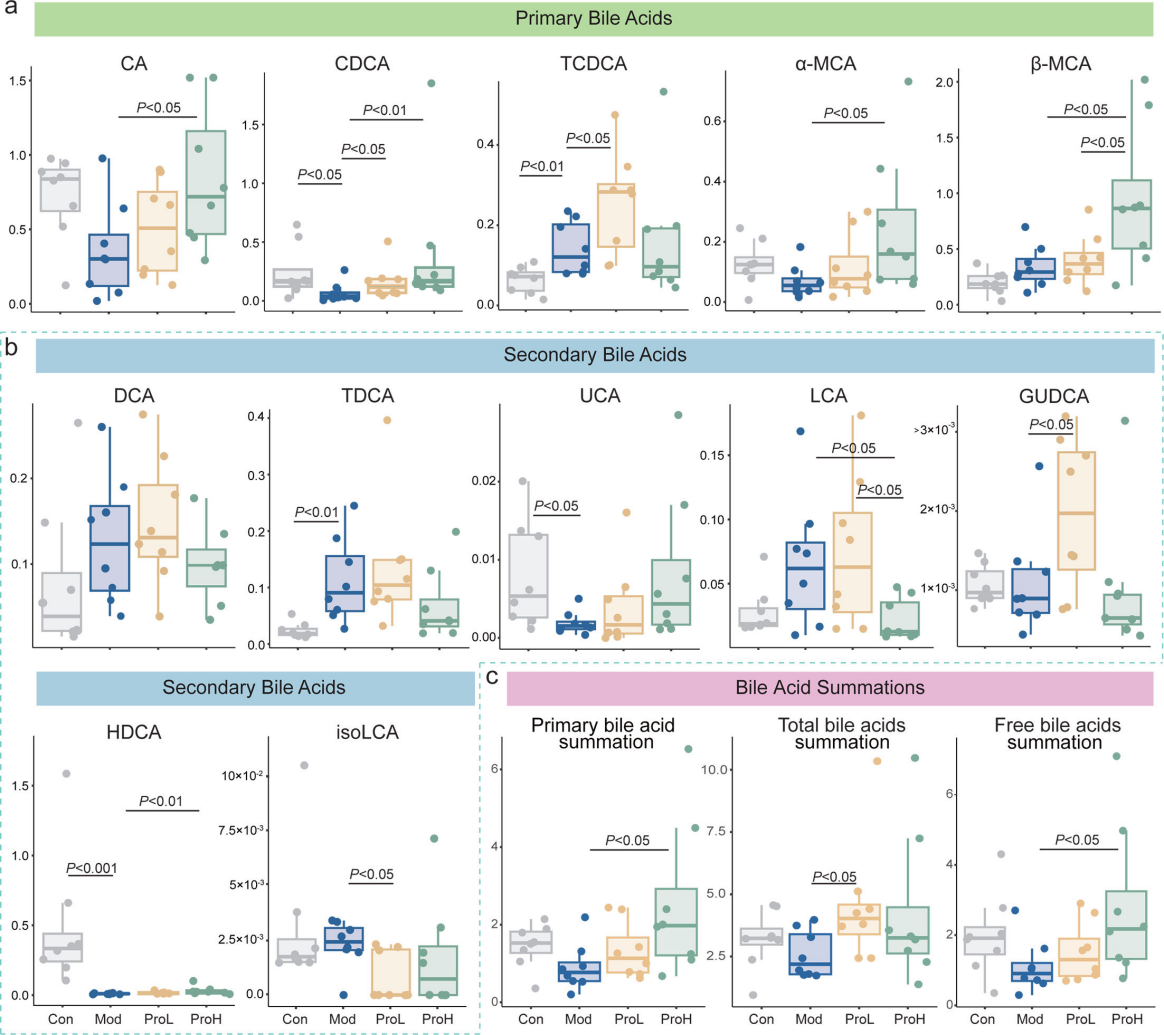
Changes in major bile acids in the serum of T2DM rats. Changes in primary bile acids (**a**), secondary bile acids (**b**), and the sum of primary bile acids, free bile acids, and total bile acids (**c**) in the Con, Mod, ProL, and ProH groups are shown. Data are presented as the means ± SEM (*n* = 8).

Bile acids and short-chain fatty acids serve as pivotal messengers in the interplay between the gastrointestinal tract and the liver, exerting a multitude of regulatory functions in the pathophysiology of T2DM (37). A targeted metabolomics approach was employed to assess the intensity of metabolite responses following probiotic intervention, based on the differential expression of microbial active metabolic modules and carbohydrate-active enzymes. A comparison of the Con group with the Mod group revealed a significant reduction in CDCA (*P* < 0.05). A similar trend was observed in CA and α-MCA, although it did not reach statistical significance. In contrast, taurochenodeoxycholic acid (TCDCA) was significantly increased after modeling (*P* < 0.05), and a comparable trend was noted in β-MCA. The primary bile acids consistently increased following probiotics intervention, with the greatest effect observed in the ProH group. Notably, TCDCA exhibited a notable decline following ProH intervention (Fig. 4a; Table S5).

Furthermore, the content of secondary bile acids, specifically GUDCA, demonstrated a statistically significant increase following the administration of the ProL intervention. In contrast, the increments in DCA, TDCA, UCA, LCA, and HDCA were less pronounced. Following ProH intervention, a notable decline was observed in the levels of DCA, TDCA, LCA, and GUDCA, while HDCA showed a marked increase (*P* < 0.05; Fig. 4b; Table S5). Interestingly, the isoLCA content was lower following probiotics intervention than in the Con group. It is noteworthy that both primary and secondary bile acids in the ProH group exhibited a closer alignment with the Con group after the intervention (Fig. 4b; Table S5). In addition, ProH significantly increased the contents of primary bile acids and free bile acid (Fig. 4c). Regarding short-chain fatty acids, the diabetes model showed a significant reduction in acetic acid content, while ProH exhibited a significant alteration in this trend. Interestingly, the modeling process also exerted a slight influence on propionic acid content. The administration of ProH resulted in a significant elevation in the concentration of propionic acid in the fecal matter of rats (Fig. S5). These results of the metabolomic analysis provide evidence that probiotics treatment produces specific physiological effects in rats.

### Probiotics modulate the transcription of genes related to inflammatory processes of the colon in rats

**Fig 5:**
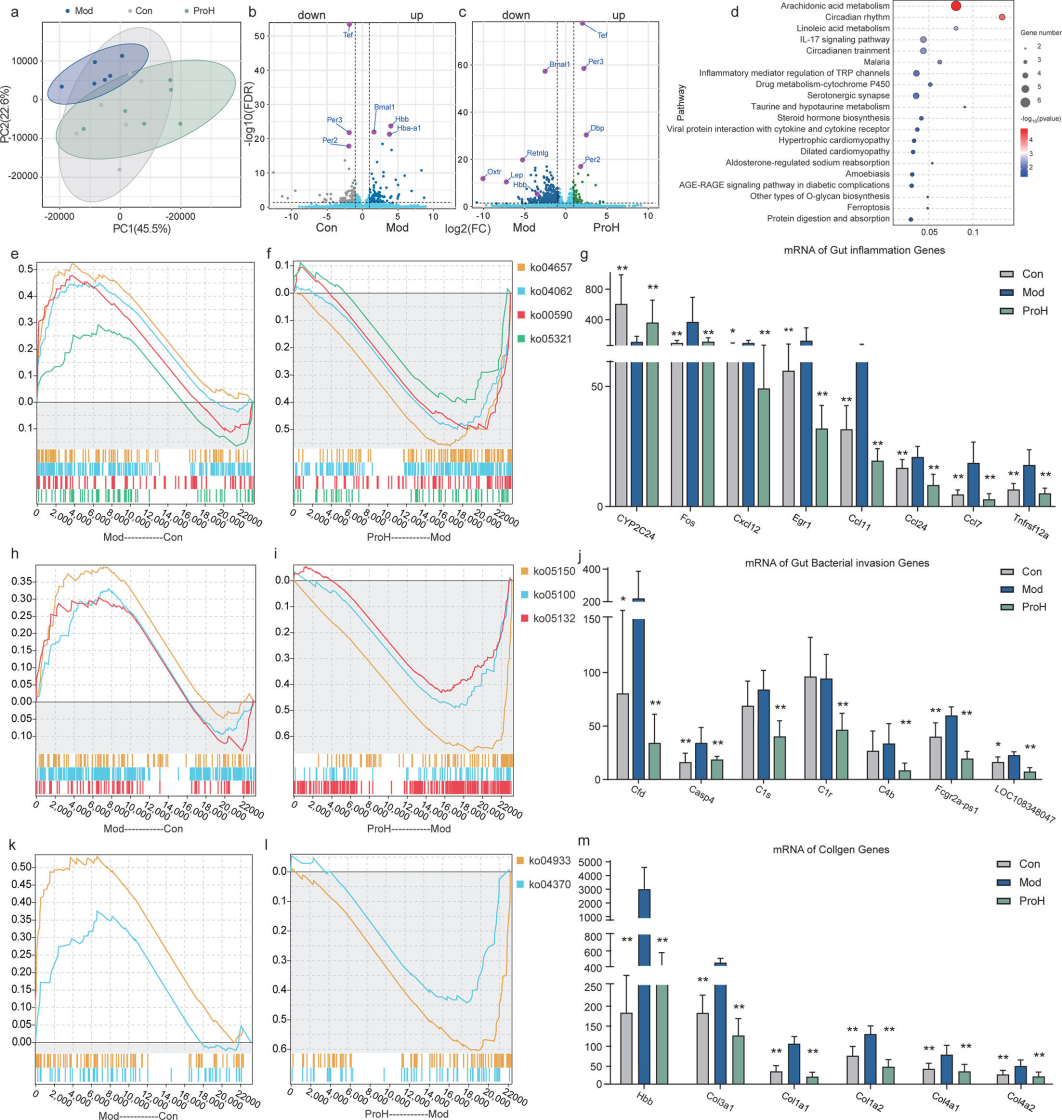
Effects of high-dose probiotics on gut transcriptome. (**a**) PCA score plot showed the clustering changes of intestinal genes in the Con, Mod, and ProH groups. Volcano plot analysis of significantly different genes in (**b**) Con versus Mod and (**c**) Mod versus ProH. Top 20 Kyoto Encyclopedia of Genes and Genomes (KEGG)-enriched pathway analyses (**d**). Gene set enrichment analysis (GSEA) of gut inflammation pathways (**e and f**) and mRNA level of related key genes (**g**). GSEA enrichment of gut pathogen infection pathways (h and i) and mRNA level of related key genes (**j**). GSEA enrichment of epidermal and tissue repair pathways (**k and l**) and mRNA level of related key genes (**m**). Data were presented as means ± SEM (*n* = 6). *P < 0.05; **P < 0.01.

The gut microbiota and the metabolites, including SCFAs, tyrosine, secondary bile acids, tryptophan, and branched-chain amino acids (38), interact with intestinal tissues, thereby affecting systemic health through the intestine’s extensive connections with the rest of the body (12). Therefore, a transcriptomic analysis was conducted to elucidate the mechanism of action of probiotics within colonic tissue, with the ProH treatment demonstrating superior efficacy. Consequently, subsequent investigations were focused on the ProH group. The PCA results revealed the most pronounced differentiation between ProH and Mod among all three groups (Fig. 5a). Of the 12,934 genes identified across the groups, 112 differentially expressed genes (DEGs) were identified with a threshold of |fold change| > 2.0 and *P* < 0.05 (Fig. S2). In comparison to the Con group, the Mod group showed higher false discovery rate (FDR) values for downregulated genes, including *Tef*, *Per2*, and *Per3*, and upregulated genes such as *Bmal1*, *Hba-a1*, and *Hbb.* These changes were reversed in the ProH group, as shown in the volcano plot (Fig. 5b and c).

A total of 112 common DEGs were selected for Kyoto Encyclopedia of Genes and Genomes (KEGG) analysis, which revealed enrichment in pathways related to arachidonic acid metabolism, circadian rhythm, linoleic acid metabolism, IL-17 signaling pathway, and circadian entrainment (Fig. 5d). Chronic inflammation is a typical contributing factor to the development of abnormal glucose and lipid metabolism (39, 40). Thus, a gene set enrichment analysis (GSEA) was conducted on the inflammatory pathways of ko00590 (arachidonic acid metabolism), ko05231 (inflammatory bowel disease), ko04657 (IL-17 signaling pathway), and ko04062 (chemokine signaling pathway). The ProH group was observed to reverse the upregulation of inflammatory genes induced by modeling in the chemokine signaling pathway (Fig. 5e and f), including *Fos*, *Cxcl12*, *Egr1*, *Ccl24*, *Ccl7*, and *Tnfrsf12a* genes (*P <* 0.05/0.01; Fig. 5g). Given the pivotal role of intestinal pathogens in inflammation, GESA analysis was performed and revealed that the ProH group reversed the upward trend of ko05100 (bacterial invasion of epithelial cells), ko05150 (staphylococcus aureus infection), and ko05132 (salmonella infection), which was induced by modeling. Specifically, transcriptional changes in genes such as *Cfd*, *Casp4*, *C1s*, *C1r*, *C4b*, *Fcgr2a-ps1*, and *LOC108348047*, resulting from the modeling, were improved (Fig. 5h through j).

Hyperglycemia can induce microcirculatory disturbances in epithelial tissues, leading to cellular inflammation and triggering epithelial repair mechanisms. Thus, a GESA analysis was conducted on ko04933 (AGE-RAGE signaling pathway in diabetic complications) and ko04370 (VEGF signaling pathway), which showed increased transcription levels of relevant genes in the Mod group, including *Col3a1*, *Col1a1*, *Col1a2*, *Col4a1*, and *Col4a2* (*P <* 0.05/0.01). Conversely, the ProH group exhibited a reversal of this trend (Fig. 5k through m). In the circadian rhythm-related pathways, the Mod group exhibited expression trends inverse to both the Con (NES-1.68, *P* < 0.01) and ProH (NES-1.57, *P* < 0.05) groups (Fig. S2b and c). Gene expression profiling revealed that the ProH group reversed the modeling-induced changes in circadian rhythm-associated genes, including *Rorc*, *Dbp*, *Bmal1*, *Dgat2*, *Tef*, *Nr1d2*, *Per1*, and *Per3* (*P* < 0.01; Fig. S2d and e).

### Probiotics modulate the transcription of genes associated with glucose, lipid, and bile acid metabolism of the liver in rats

**Fig 6:**
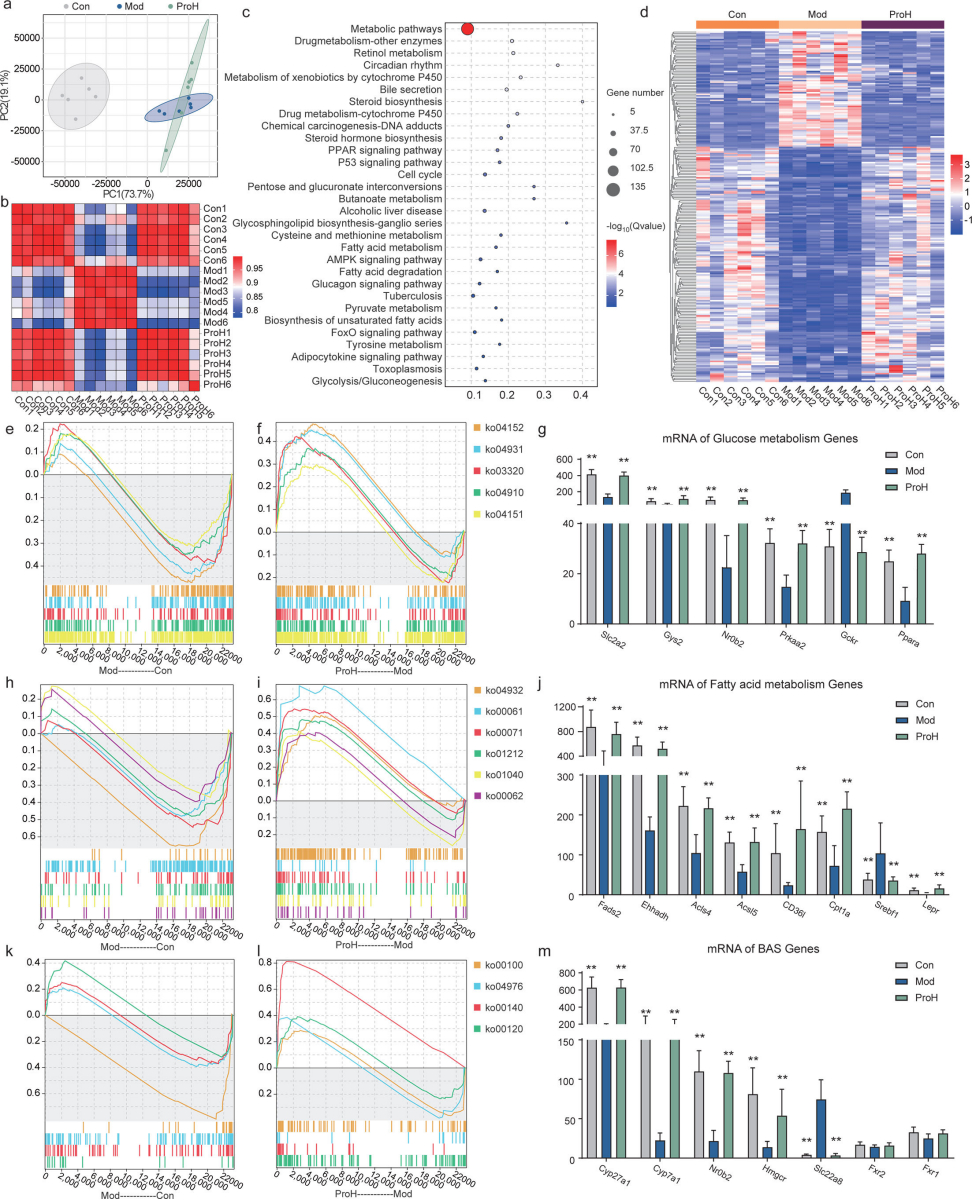
Changes of rat liver transcriptome after high-dose probiotics intervention. (**a**) PCA score plot showed the clustering changes of liver genes in the Con, Mod, and ProH groups. (**b**) Heat map of the correlation between the three groups. (**c**) Top 30 KEGG-enriched pathway analyses. (**d**) Heat map analysis of key pathway gene expression. GSEA enrichment of glucose-related pathways (**e and f**) and mRNA level of related key genes (**g**). GSEA enrichment of lipid metabolism-related pathways (**h and i**) and mRNA level of related key genes (**j**). GSEA enrichment of cholesterol metabolism-related pathways (**k and l**) and mRNA level of related key genes (**m**). Data are presented as means ± SEM (*n* = 6). *P < 0.05; **P < 0.01.

The liver, a central organ in glucose and lipid metabolism, plays a pivotal role in the progression and management of T2DM. Previous research has demonstrated that probiotics can exert a beneficial effect on chronic metabolic disorders by modulating the gut-liver axis (39, 41). In this study, H&E staining revealed a greater number of lipid droplets and lymphocytic infiltration in the livers of rats in the Mod group, whereas ProL and ProH reduced hepatic lipid accumulation and lymphocytic infiltration. Concurrently, in the iWAT, only a slight increase in adipocyte size was observed in the Mod group (Fig. S3a and b). In light of these findings, pathological assessments and transcriptomic analyses were performed on the livers of rats to elucidate the underlying mechanisms. The PCA revealed that the Mod group was significantly disparate from the other two groups (Fig. 6a). The correlation analysis between the three groups showed that the ProH group exhibited a stronger correlation with the Con group, with the majority of correlation coefficients being ≥0.95 (Fig. 6b).

A subsequent analysis identified DEGs with consistent differential expression across all groups. By applying a threshold of |fold change| > 2.0 and *P* < 0.05, a total of 1,568 DEGs were identified, with 933 DEGs being found commonly differentially expressed among all three groups. To refine this further, a filter was used to identify DEGs with inverse expression patterns between the ProH and Mod groups. This approach facilitated the curation of a distinct gene set for KEGG functional annotation. Our analysis revealed that metabolic pathways were the most significantly enriched among the identified signaling pathways (Fig. 6c). Furthermore, 170 DEGs derived from KEGG-enriched pathways were filtered out for heatmap construction. These pathways are implicated in a number of biological processes, including metabolic pathways, bile secretion, steroid biosynthesis, alcoholic liver disease, fatty acid metabolism, fatty acid degradation, the PPAR signaling pathway, the AMPK signaling pathway, and the glucagon signaling pathway. The heatmap indicated a significant divergence in gene expression profiles between the Mod group and the other two groups. Notably, the gene expression patterns of the ProH group exhibited a closer alignment with those of the Con group (Fig. 6d).

A GESA analysis was conducted on pathways associated with glucose metabolism, lipid metabolism, and cholesterol metabolism. The expression of *Slc2a2*, *Gys2*, *Nrob2*, *Prkaa2*, and *Ppara* (*P <* 0.05/0.01), the genes related to ko04152 (AMPK signaling pathway), ko03320 (PPAR signaling pathway), ko04910 (insulin signaling pathway), and ko04151 (PI3K-Akt signaling pathway), demonstrated a downward trend in the Mod group. This trend was reversed in the ProH group (Fig. 6e through g). In lipid metabolism, the expression of genes involved in pathways such as ko04932 (non-alcoholic fatty liver disease), ko00061 (fatty acid biosynthesis), ko00071 (fatty acid degradation), ko01212 (fatty acid metabolism), ko01040 (biosynthesis of unsaturated fatty acids), and ko00062 (fatty acid elongation) was elevated in the Mod group. This was evidenced by the increased transcriptional levels of *Fads2*, *Ehhadh*, *Acsl5*, *Acsl4*, *CD36L1*, *Cpt1a*, *Srebf1*, and *Lepr* genes (*P <* 0.05/0.01). The ProH group demonstrated a reversal of this trend (Fig. 6h through j). In cholesterol metabolism, the transcription levels of genes associated with bile acid synthesis and secretion, such as ko00100 (steroid biosynthesis), ko00120 (primary bile acid biosynthesis), ko04976 (bile secretion), and ko00140 (steroid hormone biosynthesis), exhibited a decline in the Mod group. Conversely, the ProH group was observed to reverse this downward trend (Fig. 6k and l). Specifically, following probiotic intervention, the expression levels of *Cyp27a1*, *Cyp7a1*, *Nr0b2*, *Hmgcr*, and *Slc22a8* (*P <* 0.05/0.01) were closer to those observed in the Con group (Fig. 6m).

### The correlation between key species and differential metabolites with blood glucose and blood lipids

**Fig 7:**
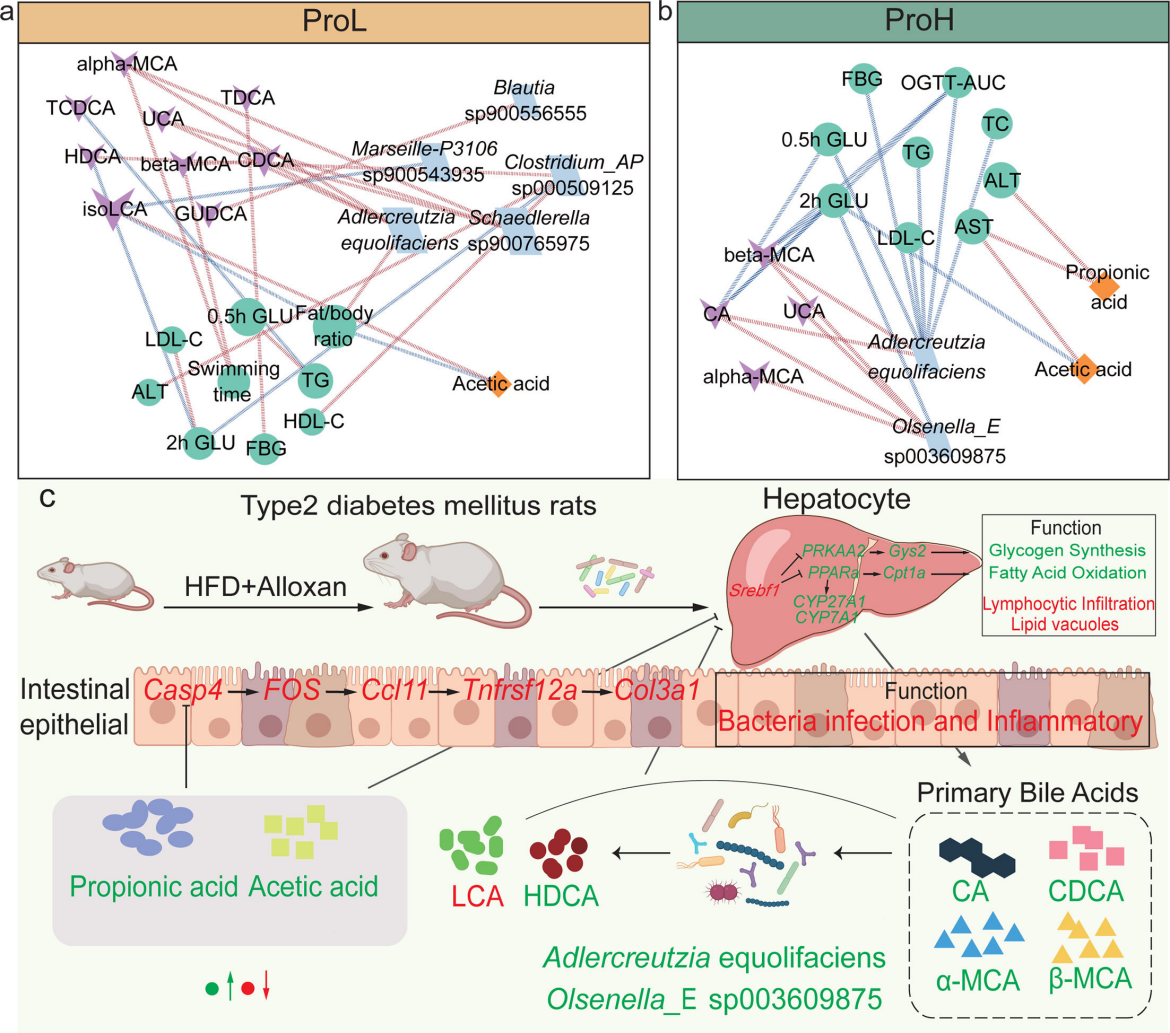
Correlation network and proposed overall mechanism of probiotics on alleviating T2DM. (**a and b**) Correlation network based on Spearman rank correlation coefficients between SGBs, differential metabolites, glycemic and lipid indices in ProL and ProH. (**c**) The proposed overall mechanism of probiotics in alleviating T2DM. The size of the nodes is proportional to the average abundance. The color of the line represents the correlation relationship. Blue, significant negative (*r* < −0.5, *P* < 0.05); red, significant positive (*r* > 0.5, *P* < 0.05).

A correlation analysis was performed on the differential SGB with metabolites, blood glucose, and lipid-related indicators in response to probiotics intervention (Table S6). A differential analysis was conducted to identify bacteria, metabolites, and blood glucose- and lipid-related indicators with significant correlations (*P* < 0.05, |*r*| > 0.5). The resulting data were used to construct a network, as illustrated in Fig. 7a and b. The data indicated that *Schaedlerella* sp900765975 and *Adlercreutzia equolifaciens* exhibited the most significant correlation with metabolites in the ProL group. Specifically, a significant positive correlation was observed between CDCA, α-MCA, and UCA (*P* < 0.05, *r* > 0.5). *Clostridium*_AP sp000509125 was more closely associated with indicators of blood sugar and blood lipid. It is noteworthy that, following ProH intervention, *Adlercreutzia equolifaciens* exhibited a statistically significant negative correlation with blood glucose and blood lipid indicators (*P* < 0.05, *r* < −0.5), whereas *Olsenella*_E sp003609875 demonstrated a statistically significant positive correlation with bile acids (*P* < 0.05, *r* > 0.5). Interestingly, the presence of *Adlercreutzia equolifaciens* was associated with disparate metabolites, glycemic, and lipid profiles in both groups. It is proposed that variations in probiotic dosage may influence the composition of the gut microbiota, which in turn affects glycemic and lipid indices, as well as metabolite levels. *Adlercreutzia equolifaciens* has been identified as a potential key target in this process.

## DISCUSSION

The intricate relationship between the gut microbiome and chronic diseases, particularly T2DM, has garnered significant attention in recent years (42). While probiotics show promise for improving glycemic and lipid dysregulation, inconsistencies across studies and limited mechanistic insights into microbiota-host crosstalk have hindered progress (42, 43). Prior work has linked composite probiotics to diabetes amelioration via gut microbiota and bile acid modulation (44), yet comprehensive analyses integrating gut-liver transcriptional networks remain scarce. Our multi-omics investigation bridges this gap by delineating how probiotics reshape microbial metabolites, intestinal gene expression, and hepatic signaling pathways to restore metabolic homeostasis in T2DM.

To address these limitations, the present study employed a multi-strain probiotic intervention in diabetic rats, examining fecal microbiota, SCFAs, serum bile acids, and the transcriptomes of the gut and liver. Our findings indicate that, in diabetic rats, multi-strain probiotic intervention improved key metabolic parameters, including OGTT-AUC, FBG, and lipid profiles (TC, TG, and LDL-C), independent of caloric intake (Fig. S1b). These results align with previous research (45, 46).

These improvements coincided with fecal microbiota restructuring, with increased microbial diversity and altered microbiota composition. While no significant differences were observed in alpha diversity, the ProH intervention markedly improved beta diversity. Notably, the relative abundance of *Adlercreutzia equolifaciens* and *Olsenella*_E sp003609875 significantly increased after ProH intervention. *Adlercreutzia equolifaciens* is known for its anti-inflammatory properties, which may be mediated through inhibition of the NF-κB pathway in human intestinal epithelial cells and hepatocytes (47). Additionally, prior work has shown that supplementation with a probiotic-fermented blueberry juice can promote an increase in *Olsenella* sp. abundance (48), which was negatively correlated with obesity induced by a high-fat diet (49). Functional metagenomic analysis further demonstrated that probiotic intervention enhanced microbial capacities for both SCFA synthesis and degradation pathways, alongside notable shifts in CAZyme profiles, particularly GHs, GTs, and CEs. Fecal SCFA analyses confirmed that ProH intervention enhanced gut microbial carbohydrate metabolism, as reflected in elevated levels of acetic acid, propionic acid, and total SCFAs. Short-chain fatty acids have been shown to positively impact the outcome of diabetes management (50). These changes are critical for modulating carbohydrate and lipid metabolism, thereby directly influencing microbiota-host metabolic interactions (51, 52).

High-fat diets can disrupt the integrity of gut microbiota, leading to the translocation of harmful bacteria across the intestinal epithelium. This translocation triggers systemic chronic inflammation, exacerbating insulin resistance and T2DM pathogenesis (37, 53, 54). In the present study, we found that oral administration of high-dose probiotics significantly restructured gut microbiota composition and downregulated the colonic expression of several inflammation-related genes, including *Casp4*, *FOS*, *Ccl11*, and *Tnfrsf12a*. For example, the cysteine protease encoded by the *Casp4* gene is a key biomarker for *Salmonella* infection (55). Its suppression indicates that probiotic intervention may help mitigate colonic mucosal damage and dysbiosis associated with T2DM, aligning with previous findings (56, 57). Furthermore, the ProH intervention also modulated the intestinal expression of circadian rhythm-related genes, highlighting their systemic regulatory potential in reversing adverse effects induced by high-fat diet administration (58–60).

In patients with T2DM, there is often an abnormal increase in hepatic gluconeogenesis or a deficiency in the timely conversion of excess blood glucose into glycogen, predisposing individuals to dysregulation of glucose and lipid metabolism (61, 62). Our study found that oral administration of probiotics downregulated the nutritional regulatory element *SREBP1c* gene and modulated the AMPK and PPAR signaling pathways. Specifically, within the AMPK signaling pathway, the upregulation of *PRKAA2* gene expression promoted the expression of the *GYS2* gene, which facilitates the conversion of glucose to glycogen, thereby enhancing carbohydrate metabolism (63). Interestingly, rats that received probiotic intervention exhibited increased swimming endurance, likely due to elevated glycogen levels in hepatocytes (64). Additionally, in the PPAR signaling pathway, the upregulation of the *PPARa* gene stimulated the expression of the *Cpt1a* gene, which enhances fat oxidation and energy expenditure (65).

Moreover, oral probiotics upregulated bile acid synthesis genes in hepatocytes, such as *CYP7A1* and *CYP27A1*, promoting the synthesis of bile acids from cholesterol, which is consistent with the increased serum levels of total primary bile acids, including CA, CDCA, α-MCA, and β-MCA. Bile acids are recognized as important regulators of glucose metabolism, influencing the progression of T2DM (15, 40, 66, 67). Notably, a previous study has shown that serum CA levels are negatively correlated with the risk of developing T2DM (68). Serum bile acids can improve carbohydrate and lipid metabolic balance in hepatocytes through the G protein-coupled bile acid receptor, thereby ameliorating diabetes (69, 70). Integrative correlation analysis among gut microbiota, serum bile acids, and blood glucose indicators in the ProH group revealed that the abundance of *Adlercreutzia equolifaciens* and *Olsenella*_E sp003609875 positively correlated with serum CA, α-MCA, and β-MCA levels. Conversely, serum CA and β-MCA levels exhibited a negative correlation with OGTT-AUC, indicating glycemic dysfunction. Thus, we propose a dual mechanism: (i) probiotic-enriched taxa augment SCFA production (acetate, propionate), reducing intestinal inflammation; and (ii) hepatic bile acid synthesis primes AMPK/PPAR pathways via enterohepatic circulation, enhancing systemic metabolism (Fig. 7c). These findings position probiotics as modulators of the gut-liver axis, offering a multifaceted strategy for T2DM management (69, 70).

While this study advances understanding of probiotic mechanisms in T2DM, key limitations warrant consideration. First, the use of a multi-strain formulation precludes assessment of individual probiotic contributions. Second, although glucose control and SCFA control levels in the ProH group were superior to the ProL group, no significant differences in the intestinal microbiota and bile acid profiles between the two groups showed that SCFAs might dominate the metabolism results. Third, the 8-week intervention period did not include intermediate monitoring of fasting blood glucose or energy expenditure, leaving the timing of probiotic effects and their metabolic cost unresolved. Finally, the transcriptome analysis discarded the ProL group because the ProH group showed superior effects on glycemic and lipid profiles, highlighting the need for comprehensive dose-response investigations to fully characterize probiotic efficacy. Addressing these gaps will refine probiotic formulations and dosing strategies for clinical translation.

### Conclusion

In summary, this study establishes SCFAs and bile acids as critical mediators bridging gut microbiota composition to hepatic carbohydrate and lipid homeostasis in T2DM. Multi-strain probiotic supplementation attenuated metabolic dysregulation by restoring microbial diversity, enhancing SCFA production, and stimulating BA synthesis, thereby improving glycemic control and lipid profiles. These improvements were mechanistically linked to AMPK/PPAR pathway activation and suppression of inflammatory signaling, underscoring the therapeutic potential of probiotics for modulating microbial-host metabolic crosstalk. Our findings position probiotics as a viable strategy for rebalancing gut-liver axis dysfunction in T2DM management, with implications for targeting microbial metabolites in metabolic therapeutics.

## MATERIALS AND METHODS

### Animals, T2DM model, and probiotics

Male specific pathogen-free (SPF) Sprague-Dawley rats (weight 130–170 g) were purchased from Zhuhai Bestest Biotechnology Co., Ltd., and maintained in the SPF-grade environment. The rats were housed in alternating light and dark cycles at a temperature of 22°C ± 2°C, humidity of 60% ± 10%, and differential pressure of ≥10 Pa. T2DM was induced by feeding the rats a high-fat diet combined with low-dose tetroxapine administration, resulting in insulin resistance and T2DM development. The multi-strain probiotics used in this study consisted of *Limosilactobacillus fermentum* LFPerfectus001, *Lacticaseibacillus rhamnosus* LRPerfectus158, *Lactiplantibacillus plantarum* LPPerfectus001, and *Bifidobacterium animalis* subsp. *lactis* BAPerfectus006, with a viable bacterial count content of 1.7 × 10^10^ CFU/g, provided by Perfect (Guangdong) Daily Products Co., Ltd.

### Experimental design

Following a 5-day acclimatization period, the rats were divided into the control (Con, *n* = 10) and the high-fat group (*n* = 30). The control group was provided with a maintenance diet, while the high-fat group received a high-fat diet. Both groups had free access to food and water. After 7 days of dietary intervention, animals displaying obesity resistance (20% with slight weight gain) were excluded based on weight gain criteria. The remaining high-fat group rats were further divided into three groups: the model group (Mod), the low-dose probiotics group (ProL), and the high-dose probiotics group (ProH) (Fig. 1a). The Con group was provided with normal chow and a daily gavage of 1 mL/100 g BW sterile water. The Mod group was given a high-fat diet and a daily gavage of 1 mL/100 g BW sterile water. The ProL and ProH groups were provided with a high-fat diet daily gavage of 2.6 × 10^9^ CFU/kg BW and 5.1 × 10^9^ CFU/kg BW probiotics, respectively, with a gavage volume of 1 mL/100 g BW. On the 57th day, alloxan was injected at a dose of 105 mg/kg BW after fasting for 24 h (without water). The high-fat diet and daily probiotic gavage were continued until the animals were sacrificed.

### Weight-bearing swimming test

On day 52, all animals underwent weight-bearing swimming experiments. Each rat was fitted with a lead weight equivalent to 5% of its body weight, attached to its tail, and placed in a swimming box to start swimming. The water depth was maintained at 30 cm, and the temperature was kept consistent with room temperature. Once the rats had reached the point of exhaustion and could no longer float, they were immediately retrieved from the water and returned to their cages. The time elapsed from the commencement of the swimming trial to the point of exhaustion was recorded as the time of the weight-bearing swimming test.

### Oral glucose tolerance test

The OGTT was carried out on day 63. After fasting for 12 h (without water), the fasting blood glucose level (0 h blood glucose value) was measured. Following the administration of the test substance via gavage over a period of 20 min, the rats in each group were given an oral glucose load of 2.5 g/kg BW. Blood glucose levels at 0.5 and 2 h after glucose administration were determined, and the AUC for blood glucose at 0, 0.5, and 2 h was calculated to assess the oral glucose tolerance of the rats.

### Sample collection

Fecal samples were collected from the rats on day 62. Naturally excreted fecal samples were collected under sterile conditions using sterile EP tubes placed at the anus of the rats. The rats were anesthetized on day 64, and their eyes were removed to collect blood (2–5 mL), which was centrifuged to obtain serum. The fecal and serum samples were stored at −80°C until use. For tissue samples, the fat around the testis and kidneys was weighed separately, and liver and colon tissues were pre-cooled in liquid nitrogen before being stored at −80°C for subsequent transcriptome analysis.

### Fecal metagenome sequencing

DNA samples were extracted from fecal samples using the QIAamp Rapid DNA Fecal Extraction Kit (QIAGEN, Germany) according to the manufacturer’s instructions. The quality of the extracted DNA was assessed using 1% agarose gel electrophoresis, requiring a DNA concentration of >20 ng/µL and an absorbance ratio of 260 nm/280 nm within the range of 1.8–2.0. DNA samples meeting these quality criteria were used to construct libraries according to the instructions of the NEBNext Ultra DNA Library Construction Kit (New England Biolabs, USA). A 16-base random sequence was indexed by PCR under the following cycling conditions: 95°C for 3 min; 12 cycles of 98°C for 20 s, 58°C for 30 s, 72°C for 30 s; followed by a final extension at 72°C for 5 min.

The prepared DNA libraries were sequenced on the Illumina NovaSeq platform by Novogene Technology Co., Ltd. (Beijing, China). A total of 32 fecal samples were sequenced, generating 204.2 Gbp of data after quality control. The average depth of sequencing coverage per sample was 6.38 Gbp (Table S7), and the total sequencing volume for all samples was sufficient to meet the needs of subsequent analysis. MetaBAT2, VAMB, SemiBin, and MetaDecoder were used for metagenome binning, resulting in 2,119, 1,220, 2,473, and 2,017 bins, respectively. Das_Tool was used to merge and refine these original bins, yielding 1,115 bins. Further classification using CheckM according to the latest standards identified 929 high-quality genomes. After de-redundancy, 189 high-quality SGBs were obtained, corresponding to 11 phyla, 14 classes, 24 orders, 35 families, 112 genera, and 161 species.

### Serum bile acid analysis

Bile acid standards were accurately weighed to prepare a concentrated stock solution at 1,000 µg/mL in methanol. This stock solution was diluted with 30% methanol to generate a series of 10 standard curve points. All stock and working solutions of standards were stored at −20°C. Serum samples were mixed with 600 µL of methanol prechilled to −20°C in a centrifuge tube and centrifuged at 12,000 × *g* for 10 min at 4°C. The supernatant (400 µL) was evaporated to dryness using a vacuum concentrator, and the residue was reconstituted with 100 µL of 30% methanol. The reconstituted solution was filtered through a 0.22 µm membrane, and the filtrate was transferred to vials for liquid chromatography-mass spectrometry analysis. Quantification of bile acids was performed using an EXion LC liquid chromatograph and an AB6500 Plus mass spectrometer (AB SCIEX, USA). The targeted metabolic analysis of bile acids was outsourced to PANOMIX Biotech Co., Ltd. in Suzhou, China.

### Fecal SCFA analysis

Fecal samples were accurately weighed (to the nearest 0.001 g) and transferred to EP tubes. A phosphoric acid solution (1.0% [vol/vol], 0.75 mL) was added along with a steel bead, and the mixture was vortexed thoroughly before being subjected to ultrasonication on ice for 10 min. The samples were then centrifuged at 12,000 rpm and 4°C for 10 min. A 200 µL aliquot of the supernatant was transferred to a new EP tube, followed by the addition of 500 µL of methyl tert-butyl ether. The mixture was vortexed and ultrasonicated on ice for another 10 min. A second centrifugation at 12,000 × *g* and 4°C for 10 min was performed, and 200 µL of the resulting supernatant was collected for analysis. Gas chromatography detection conditions were based on the method described by Shi et al. (71).

### Liver and intestinal transcriptome sequencing

Transcriptomics analysis was performed as previously described (72). Briefly, total RNA was extracted from liver and intestinal tissues using the Trizol reagent kit (Invitrogen, USA) following the manufacturer’s instructions. Sequencing-ready cDNA libraries were prepared and paired-end sequenced on the Illumina Novaseq6000 platform by Gene Denovo Co. (Guangzhou, China). Raw RNA sequencing reads underwent quality filtering, followed by transcriptomic processing and differential expression analysis using R Bioconductor tools and the DESeq2 package (73). Differentially expressed genes were identified using thresholds of *P* < 0.05 and |fold change| > 1.5; these genes were selected for subsequent analyses.

### Statistical analysis

All statistical analyses were performed using the R software (version 4.1.2). To evaluate the microbiological results, PCoA and multiple R packages, including vegan, optparse, mixOmics, ggplot2, and ggpubr, were used for detection and visualization. *P*-values were calculated using the Adonis test based on 999 permutations. The differences between the groups were assessed using the Wilcoxon rank-sum test, with the *P*-values adjusted for multiple comparisons using the Benjamini-Hochberg procedure. A *P-*value of less than 0.05 was considered statistically significant. All graphical representations were generated using R software and Adobe Illustrator.

## Data Availability

The metagenomics data that support the findings of this study have been deposited in the CNGB Sequence Archive of China National GeneBank DataBase (CNGBdb; https://db.cngb.org/cnsa/) under accession number CNP0007000 for rat metagenomics data sets.

## SUPPLEMENTAL MATERIAL

Figure S1 msystems.00369-25-s0001.eps Changes of fecal short-chain fatty acids and food intake in T2DM rats.

Figure S2 msystems.00369-25-s0002.eps Changes in colon genes and pathways after ProH intervention.

Figure S3 msystems.00369-25-s0003.eps Histopathology analysis.

Table S1 msystems.00369-25-s0004.eps Significant differences in blood glucose and lipids after intervention.

Table S2 msystems.00369-25-s0005.eps Taxonomic information of SGBs with relative abundance >0.1.

Table S3 msystems.00369-25-s0006.eps Significant difference in SGBs after molding.

Table S4 msystems.00369-25-s0007.eps SGBs with CAZyme-encoding gene identified.

Table S5 msystems.00369-25-s0008.eps The serum bile acids and fecal short-chain fatty acids were significantly changed after probiotic intervention.

Table S6 msystems.00369-25-s0009.eps Correlation analysis on the differential SGB after response to compound probiotic intervention and differential metabolites and blood glucose and lipid-related indicators.

Table S7 msystems.00369-25-s0010.eps Data size of metagenomic sequencing for rat samples.

ASM does not own the copyrights to Supplemental Material that may be linked to, or accessed through, an article. The authors have granted ASM a non-exclusive, world-wide license to publish the Supplemental Material files. Please contact the corresponding author directly for reuse.
